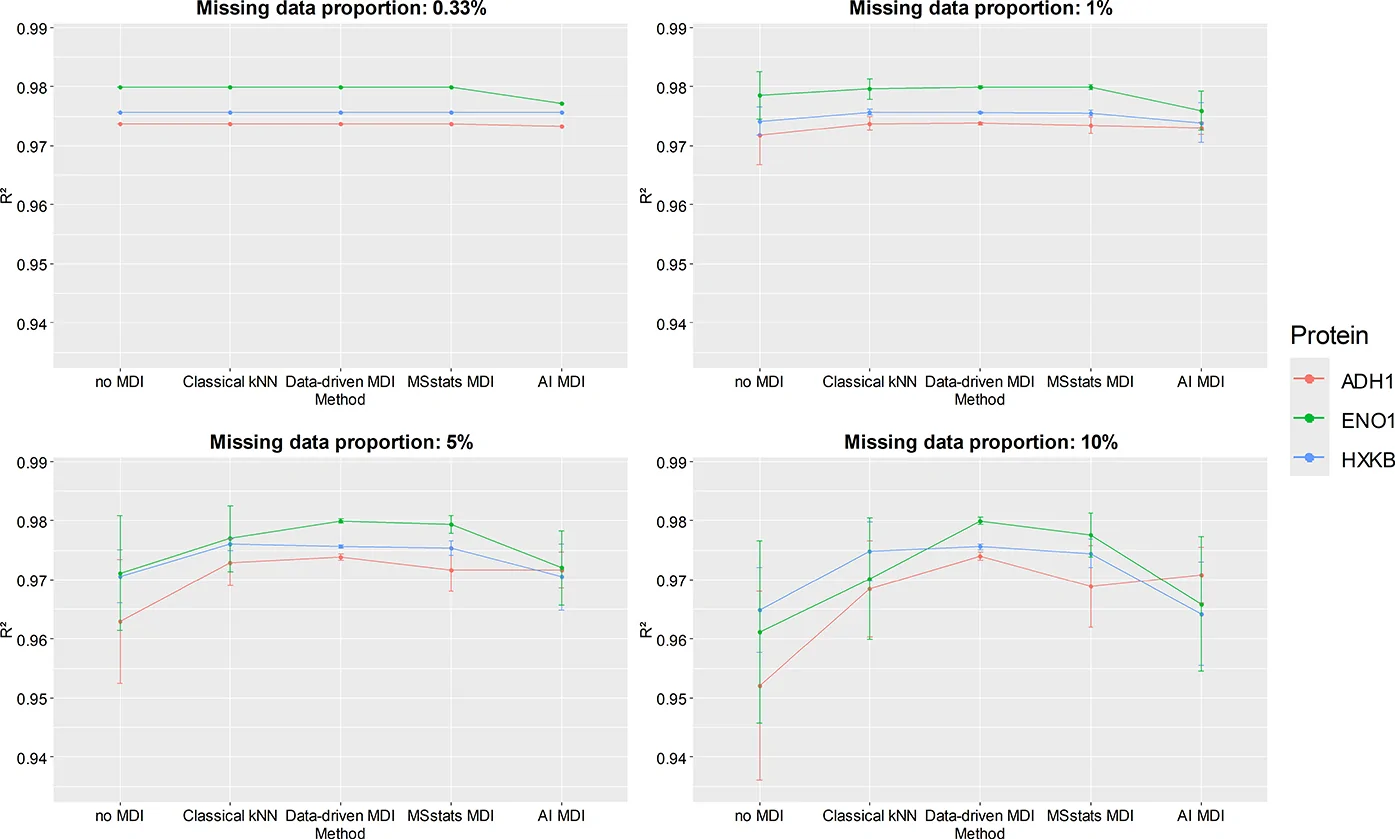# Label-Free Targeted Proteomics Data Analysis Workflow Selection – Benchmarking AI-based and Data-Driven Approaches

**DOI:** 10.1016/j.mcpro.2026.101631

**Published:** 2026-07-23

**Authors:** Daniel Fochtman, Łukasz Marczak, Monika Pietrowska, Joanna Polańska, Anna Wojakowska

**Affiliations:** 1 Laboratory of Mass Spectrometry, Institute of Bioorganic Chemistry Polish Academy of Sciences, Poznan, Poland; 2 Center for Translational Research and Molecular Biology of Cancer, Maria Sklodowska-Curie National Research Institute of Oncology, Gliwice Branch, Gliwice, Poland; 3 Department of Data Science and Engineering, Silesian University of Technology, Gliwice, Poland

**Keywords:** label-free targeted proteomics, data analysis workflow selection, AI-based approaches, data-driven approaches, prmPASEF

## Abstract

As global proteomics continues to advance, the number of identifiable proteins has increased substantially. However, this does not inherently ensure optimal quantitative performance. While targeted assays using isotope-labeled peptides can be developed, label-free strategies remain an attractive and cost-efficient option for methodological validation. Yet, systematic evaluations of data analysis workflows for label-free targeted proteomics, particularly those incorporating artificial intelligence (AI)-based tools, are still limited. Therefore, this study aimed to benchmark multiple data analysis approaches for label-free targeted proteomics as a validation framework for results obtained from global analyses. Missing-data imputation (MDI) strategies, including no MDI, k-nearest neighbors, data-driven, MSstats and AI-based methods, were evaluated, alongside consolidation and testing frameworks such as mathematical summation, best-peak selection, AI-based scaling with univariate statistics, *p*-value integration, MSstats Tukey’s median polish or linear models and multivariate testing. Data-driven MDI combined with *p*-value integration consistently showed the strongest performance across accuracy, precision, specificity, and false-discovery rate, outperforming all other strategies. These findings demonstrate that careful selection of data analysis workflows can yield substantially improved quantitative outcomes compared with commonly used approaches such as simple mathematical summation. Although our conclusions are based on a controlled benchmarking dataset comprising three yeast proteins spiked into a constant human background using only one targeted approach, they can be generally applied as a default workflow for biomarker validation using the label-free approach.

## Highlights

• Label-free PRM data analysis approaches remain undeveloped and poorly benchmarked.
• Data-driven approach outperformed other missing data imputation methods.
• *p*-Value integration resulted in best PPV, precision, specificity and accuracy.
• AI tools lacked precision, highlighting need for fit-for-purpose targeted models.

## Graphical Abstract

**Figure:**
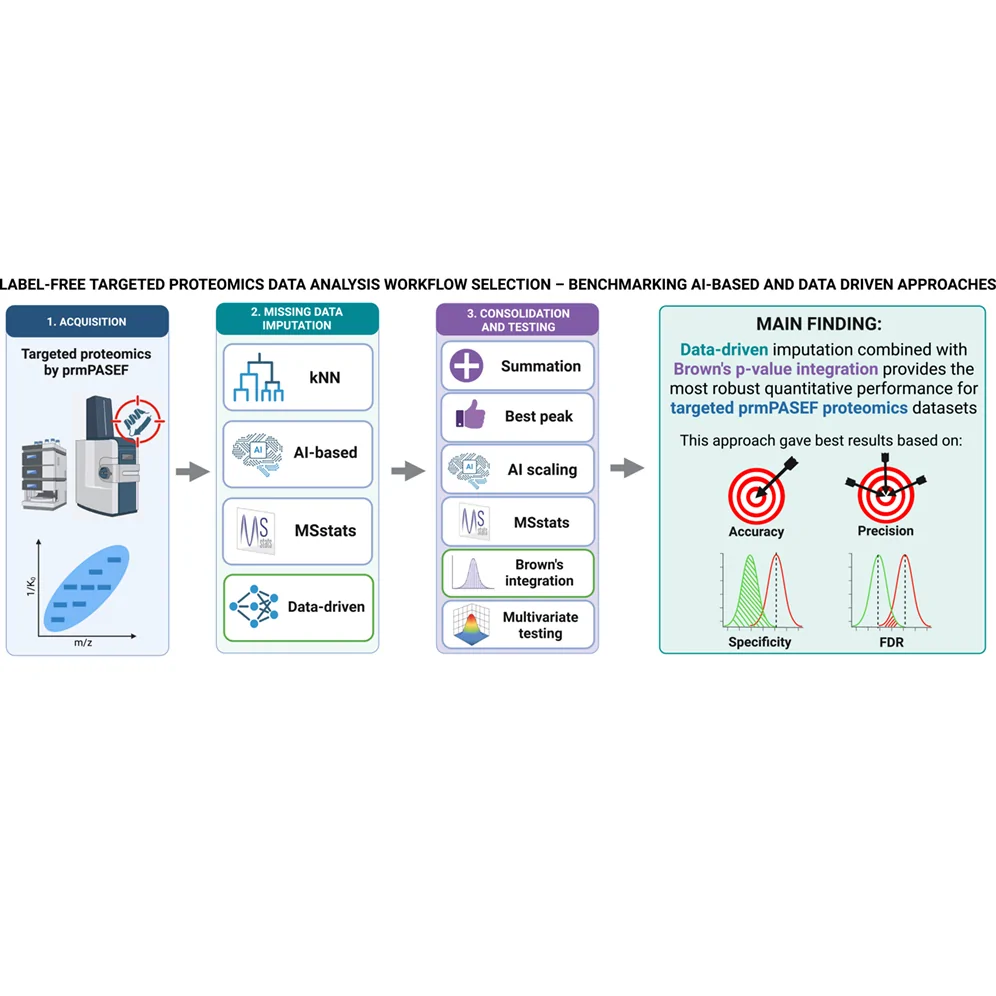

## In Brief

Label-free PRM proteomics offers an approachable strategy for validating biomarker candidates from global discovery studies, but dedicated data analysis workflows remain undeveloped and poorly benchmarked. Thus, this study compares missing data imputation, consolidation and statistical testing approaches using a controlled prmPASEF spiked-in dataset. In the end, data-driven MDI combined with Brown’s p-value integration achieved the best overall performance. Current AI-based approaches lacked the precision required in PRM, highlighting the need for models specific to targeted analyses.

Global proteomics has expanded significantly in recent years, driven by advances such as ion mobility spectrometry and faster, higher-performance mass analyzers (1, 2). As a result, data-dependent (DDA) and data-independent acquisition (DIA) strategies have become the primary tools for discovery-driven studies, enabling the identification of thousands of proteins without prior knowledge about which peptides are quantifiable or extensive method optimization. Using a label-free approach further enhances accessibility by eliminating the need for stable isotope-labeled (SIL) peptides. Yet, global analyses have their intrinsic drawbacks – they produce extremely large datasets that require automated processing, often without reference peptides, which complicates quality control. Moreover, a harsh statistical penalty is applied when a large number of comparisons is to be performed (*e*.*g*., by Benjamini-Hochberg multiple testing correction).

For experiments requiring precise quantification of a defined subset of proteins, targeted MS - most commonly achieved *via* selected, multiple, or parallel reaction monitoring (SRM, MRM, PRM) - remains the method of choice. When combined with SIL peptides, these assays offer high reproducibility and straightforward quality control, which has enabled their successful application in clinical diagnostics (3, 4, 5, 6, 7, 8). Unlike antibody-based approaches, these analyses are fast and do not suffer from problems inherent to the use of antibodies, that is cross-reactivity, lot-to-lot variability and limited multiplexing capacity (9, 10, 11, 12). However, the scalability of SIL-based methods is limited: synthesizing and spiking-in hundreds of peptides becomes prohibitively expensive, and the need to measure both endogenous and heavy-labeled versions effectively halves analytical throughput. Label-free targeted approaches provide an attractive alternative, particularly for validating protein biomarker candidates identified in global analyses. Here, omitting SIL standards increases the number of proteins that can be evaluated, while *a priori* knowledge from global analyses informs method optimization (13, 14). Although this comes at the cost of reduced quality control, label-free targeted proteomics offers advantages such as ideal chromatographic peak coverage and low operational cost.

Generally, targeted proteomics workflows have been extensively used over the past decade, with well-established analytical frameworks for both method development and data acquisition (15, 16). Foundational work has outlined the principles, advantages, and limitations of SRM and PRM quantification, including considerations related to assay design, reproducibility, and quantitative accuracy (17, 18, 19). A critical aspect of these workflows is the selection of appropriate transitions, as suboptimal method design or insufficient optimization can substantially affect quantitative performance. This has led to the development of both manual and automated strategies for transition refinement, which allow targeted analyses to achieve optimal quantitative accuracy and sensitivity with minimal data missingness, as compared with global proteomics analyses (20, 21). In parallel, some approaches for statistical analyses of label-free targeted data have been developed, including linear modeling frameworks that explicitly account for variability across runs and features – these have subsequently been incorporated into widely used tools such as MSstats (22, 23).

Despite the usefulness of the label-free targeted approach, its specific data analysis workflows remain underdeveloped, with many tools available only for global DDA/DIA approaches (24, 25, 26). Most studies rely on simple mathematical summation followed by univariate statistics (27, 28, 29, 30, 31, 32, 33, 34, 35, 36, 37, 38, 39, 40, 41), despite the availability of newer options, including artificial intelligence (AI)-based tools (42, 43). Summarization followed by testing may not necessarily provide the best results, from a statistical point of view, especially for targeted approaches where full MS2 peak chromatograms are available. Nonetheless, the performance of these workflows has not been systematically compared, leaving uncertainty about which approaches provide the most reliable results.

In the end, we hypothesized that exploiting fragment ion ratios for missing-data imputation, combined with dependence-aware *p*-value integration would outperform commonly used summation-based label-free PRM workflows. Therefore, the aim of this study was to benchmark multiple data analysis workflows for label-free targeted proteomics used as a validation framework for results obtained from global proteomic analyses.

## Experimental Procedures

### Experimental Design and Statistical Rationale

To achieve the setout goal of this study, a benchmarking dataset was generated where three yeast proteins were spiked into a constant human proteome background at known concentrations. Target peptides were selected as unique, with zero missed cleavages, and ranged from 7 to 20 amino acids, with carbamidomethylation as an allowed modification. A discovery experiment using ddaPASEF was performed, informing a final method, which encompassed 3 spiked-in yeast proteins, iRT standards, and 30 human proteins with 3 precursor peaks, each with the top 8 fragment ions ranked by intensity. To minimize batch effects, all samples were acquired within a single set of LC–MS/MS runs. Thus, the final benchmarking dataset consisted of a batch of 6 sets with differing yeast protein concentrations, each with 5 technical replicates. Additional samples were run to aid in determining the linear range, including 5 replicates of a blank. The inclusion and exclusion of datapoints are described in detail in the Results section. Samples were run from the lowest to the highest concentration of yeast proteins, thereby minimizing carryover. Normality of data distribution was checked using the Shapiro-Wilk test, then accordingly, statistical methods have been chosen to allow for non-normally distributed data (*i*.*e*., by using Mann-Whitney non-parametric test). The process of comparison of missing data imputation approaches was started by removing excessively missing data based on the results of hierarchical clustering, that is by removing data clustered in the first branch. Next, we utilized mean values as a measure of central tendency and standard deviation as a measure of dispersion. In the case of data consolidation and statistical testing comparison, multiple parameters were derived from the confusion matrix, including false-discovery rate, precision, accuracy, and specificity. Since many of the measured peptide fragments stem from the same protein, data dependence was accounted for in the *p*-value integration workflow using Brown’s procedure (44). Significance cut-offs have been set at false-discovery rate of 5% as determined by the Benjamini-Hochberg procedure.

### Sample Preparation

Post-operative material for research purposes was used under the approval of the local Ethics Committee at the National Research Institute of Oncology Branch in Gliwice, approval no. KB/430-120/21. The study was conducted in accordance with the principles outlined in the Declaration of Helsinki. The tissue donor signed an informed consent form, attesting that they had voluntarily and consciously taken part. A human rectal cancer tissue (adenocarcinoma of grade G2 and stage T3N0M0) sample was collected in 2022 during standard surgical treatment. The resected tissue was immediately snap-frozen in liquid nitrogen and stored at −80 °C until analysis in 2025. Three tissue sections taken from the edges and the center of the sample were stained with H&E and evaluated by an experienced pathologist to assess tissue composition and tumor cell content. Histopathological evaluation showed that the examined tissue consisted of 10% mucosa, 30% muscularis propria, 50% submucosa, and 10% cancer cells.

Sample was first homogenized in 1% SDC in 50 mM AmBIC with a Precellys 24 homogenizer (Bertin Technologies) and sonicated for 10 min at 4 °C and centrifuged at 11,000*g*, 4 °C for 10 min. The supernatant was collected, and the protein concentration was determined using the BCA assay (Thermo Fisher Scientific). Assuming 100% digestion efficiency, 400 ng of protein per 5 μl injection was used for subsequent sample preparation by in-solution digestion. Spike-in of three yeast proteins at concentrations ranging between 0.4 ng to 3.97 ng per 5 μl injection was added to the sample. These proteins included ADH1 (SigmaAldrich), HXKB (SigmaAldrich) and ENO1 (SigmaAldrich). The sample volume was equalized to 25 μl using 50 mM AmBIC, and 1.5 μl of 100 mM DTT was added, followed by sample heating to 95 °C for 5 min. After the sample had reached room temperature, 3 μl of 100 mM IAA was added, and the sample was incubated in darkness for 20 min. Next, trypsin (Promega) was added in an enzyme-to-protein mass ratio of 1:25, and the sample was digested for 16 h at 37 °C. SDC was disposed of by adding 1.5 μl of 10% TFA. Sample was mixed for 10 min, spun down at 11,000*g* for 7 min and the supernatant was collected. An iRT standard (Biognosys) was added as per the manufacturer’s instructions.

### LC-MS/MS Analysis

Thermo Fisher Scientific’s Dionex UltiMate 3000 RSLC nanoLC system was coupled to a Bruker timsTOF Pro mass spectrometer with a CaptiveSpray source. Peptide separation was achieved using a Bruker PepSep ULTRA (25 cm × 75 μm × 1.5 μm) C18 column kept at 50 °C with a trap-and-elute approach using the Thermo Fisher Scientific’s PepMap Neo Trap Cartridge at 60 °C. A reverse phase gradient was used, starting at 5% acetonitrile with 0.1% formic acid, increased linearly to 35% acetonitrile with 0.1% formic acid in 80 min. The standard prmPASEF (Parallel Accumulation-Serial Fragmentation) method was used with slight modifications, that is ramp time was set to 166 ms with duty cycle locked at 100% spanning 1/K0 range of 0.6 to 1.6 V⋅s/cm^2^ and m/z range of 100 to 1700. The maximum allowed MS1 scan interval was set at 1.8 s, and the collision energy increased linearly from 20 to 59 eV based on the 1/K0 value. Ion mobility window selection assumed a resolving power of 30, and an RT window of 10 min was used. The transitions monitored for each analyte are provided in Supplemental Table S1. The analysis was designed according to the third tier of targeted MS measurements as defined by *Carr et al* (18).

### Data Preprocessing

Results in the form of .d files were imported into Skyline-daily v.24.1.1.398 (45) and manually checked for integrity, including removal of interferences based on peak intensity, symmetry, and fragment co-elution. Ion match tolerance was set to 10 ppm and TIC was used as a normalization method. MS filtering options were set to use high-selectivity extraction and a TOF mass analyzer with a resolving power of 50,000. All matching scans were included. Results including peak areas, protein and peptide names, precursor and fragment m/z values and charges were exported into the R environment (version 4.4.3), which was used for subsequent analysis. The peak areas used were all TIC normalized transition peak areas that were not transformed prior to analysis in the R environment. This data is available as Supplemental Table S2. Generally, we distinguish between the representation of non-observed values in the quantitative matrix and the mechanism by which they arise. This mechanism can be described and classified in two distinct manners – using established statistical classification and based on the way Skyline software encodes the data. First, generally in statistical terms, missing data can be missing completely at random (MCAR), it can be missing at random (MAR) or not-at-random (MNAR). As an example, MNAR, is often attributed to MS-based proteomics analyses, where the measured signal falls just below the limit of detection (LOD)/limit of quantification (LOQ). Although other types of data missingness such as MCAR and MAR can also be present, in case of MNAR, missingness can be attributed to a specific mechanism. The second classification has to do with the way Skyline encodes the data matrix – when a measurement was possible, but the peak area was indistinguishable from the noise, numerical zero value is assigned. When a measurement is not possible, for example due to analysis constraints set in Skyline (*e*.*g*., when a m/z value for a given peptide fragment falls outside the set m/z range), #N/A is assigned instead. In our benchmark of missing data imputation, described in detail below, missing data in the form of #N/A values is used and generated programmatically with random selection of peptide fragment peak areas over hundreds of iterations. Therefore, the benchmark compares imputation strategies for fragment missing peak area values within otherwise detected peptide, without separately modeling MCAR, MAR or MNAR mechanisms, while still recognizing the specific way in which Skyline encodes its values.

### Missing Data Imputation Approaches

In this study, missingness was considered mainly at the transition level within an otherwise detected peptide transition group. Specifically, the evaluated scenarios included cases in which one or more fragment-ion transitions were not quantified in a given sample, for example due to low signal intensity or due to transition interference. Thus, the benchmark was designed to address the partial incompleteness of transition-level PRM data, in which at least part of the peptide-specific signal remained detectable in a given sample. In contrast, cases in which the entire peptide transition group was absent in a sample, for example, because of very low peptide abundance, chromatographic drift, or scheduled acquisition failure, were not the primary target of the present analysis and were not explicitly modeled as a separate missingness category. To this end, the selected methods of missing data imputation were:

## No Missing Data Imputation (No MDI)

### K-Nearest Neighbors (Classical kNN)

As a classical approach generally used for data imputation, not only in targeted proteomics studies, k-nearest neighbors procedure was utilized. This algorithm derives the imputed value from a weighted average of the “k” number of its nearest neighbors. In this study, a k value of 3 was chosen, with weights used for averaging corresponding to the Euclidean distance between the case and the neighboring value. This implementation was achieved using the multiUS v.1.2.3 package with its data scaling parameter enabled by default.

#### Data-Driven MDI

In the data-driven approach, peptide fragments’ (mostly of the y and b type) peak areas derived from consecutive MS^2^ scans are utilized to impute missing values. It is assumed that the ratio of these peak areas to each other remains constant across samples (46). Calculating such a ratio for all possible fragment combinations in all samples is done first. When all fragment ratios are calculated, their median and CV are established. Based on the lowest possible CV, another fragment is chosen as a “reference”. Following mathematical rearrangement with the ratio and a reference fragment peak area, missing data can be imputed simply. An expanded description of the data-driven missing data imputation algorithm is presented in Supplemental Note 1.

#### MSstats MDI

In the MSstats missing data imputation approach, the MSstats v.4.16.1 package for R was used. Briefly, this method relies on an accelerated failure time model to account for observations, assuming missing values are due to low abundances falling below the level of detection. In this framework, intensities are modeled with an account for both runs and features. Missing values are imputed by estimating their expected values based on the observed distribution of the corresponding feature across runs, as well as information from other features belonging to the same protein. Imputation is only performed when sufficient information is available (*i*.*e*., when the feature is observed in at least one run and other features are present in the same protein) and no values are imputed for proteins entirely missing within a given run.

#### AI-Based Approach (AI MDI)

The AI approach uses the same mathematical assumptions as the data-driven MDI, although the fragment ratios are not calculated from the available dataset. Instead, scaled fragment intensities are taken from an AI “*ms2pip_timsTOF2024*” model deposited in the Koina repository (42). It is then possible to calculate fragment ratios, as was the case in the data-driven approach. In this study, we utilized the model that best fit the benchmarking dataset, that is the “*ms2pip_timsTOF2024*” model (47, 48) with the normalized collision energy set at a constant value of 31. Data accession was realized programmatically using the KoinaR v.1.0.2 package. It should be noted that this model does not support doubly charged fragments; thus, they are removed from the subsequent analysis.

### Data Consolidation and Statistical Testing Approaches

#### Mathematical Summation

In this computationally straightforward consolidation method, all of the fragment peak areas are mathematically summed together. An appropriate univariate statistical testing follows, such as the non-parametric Mann-Whitney test, as many measurements available per peptide/protein have been reduced into one abundance value per sample.

#### Best Peak Selection (Global or local)

The best peak selection algorithm aims to achieve data consolidation by selecting only a single fragment to be compared using “standard” univariate testing. Here, selection is performed by finding a fragment with the highest peak area (*i*.*e*., highest signal-to-noise ratio) across samples for a selected peptide/protein, either globally or locally. In the global approach, the median peak area for each fragment is calculated first. The fragment with the highest median peak area is chosen as the best and is used for subsequent testing. In the local approach, the fragment with the highest peak area is chosen for each sample separately. The fragment that appears most frequently with the highest peak area is chosen as the best and is used in further univariate testing.

#### AI Data Scaling and Summation

This method utilizes the tools described in the missing data imputation section, specifically an AI model to scale the data prior to mathematical summation. In practice, a ratio between all possible peptide fragment peak areas is obtained and calculated from “*ms2pip_timsTOF2024*” model set at normalized collision energy of 31 which was accessed using the Koina repository (47, 48). Next, a reference fragment is chosen – either having the highest m/z value (referred to as “heaviest”) or having the highest peak area (referred to as “highest”). All fragment peak areas, besides the reference, are then rescaled using the obtained ratio and a reference fragment peak area. Scaling does not alter the data dimensionality; therefore, further consolidation and statistical testing are required. Here, mathematical summation and univariate testing were used, exactly as was described in the section “*Mathematical summation*”.

#### *P*-Value Integration

*p*-value integration is an approach that starts with univariate statistical testing for each fragment peak area separately. Since each protein/peptide is comprised of multiple fragments, many *p*-values are produced, and their straightforward comparison is not possible. Thus, *p*-value integration is performed using Brown’s method (44). This method was selected as it corrects for the data dependency between fragment peak area values – multiple fragments stem from the same peptide, thus their abundance is directly correlated. Other methods, such as the often-used Fisher’s *p*-value integration, do not account for data dependence; therefore, they were not tested. Assuming a significance level of 5%, this value is imputed into Brown’s algorithm using the correlation matrix from the data to be compared, and a corrected significance level is produced. This corrected value is then used for determining which peptides are differentially abundant. Unlike in other methods, no single abundance value is obtained (only a single *p*-value); thus, for purposes of data visualization, alternative consolidation approaches must be used (*e*.*g*., mathematical summation). This approach was implemented using the EmpiricalBrownsMethod v.1.34.0 package.

#### Multivariate Testing

Multivariate testing is an approach that considers more than one variable when performing statistical tests. In the targeted proteomics analyses, the number of peptide fragment peak areas is higher than one; thus, this approach can be utilized to test for significance, taking all these values into account. Because multiple fragment ions are measured for each peptide, fragment-level signals were analyzed jointly. Fragment ions originating from the same peptide are not separate measurements; they rather constitute correlated values influenced by the same underlying peptide concentration and shared measurement conditions. Therefore, the multivariate approach was not intended to treat fragment ions as independent variables, but to capture their collective structure within a peptide/protein. We employed a non-parametric version of the multivariate analysis of variance as the testing approach, which included a permutational analysis, known as PERMANOVA (49). In our study, we used Euclidean distance metric and set the number of permutations to 15,000 with the implementation of the algorithm realized using the vegan v. 2.6 to 10 package. Again, as is the case in *p*-value integration, no single abundance value is returned, thus other data consolidation approaches have to be used for *e*.*g*., data visualization.

#### MSstats Tukey’s Median Polish (TMP)/Linear

For these data consolidation methods, the MSstats v.4.16.1 package for the R programming language was used. Consolidation was performed using two alternative approaches: TMP and a linear model method. TMP uses an iterative median-based decomposition to estimate feature intensities across runs, thereby reducing the influence of outliers. In contrast, the linear method relies on average-based summaries. Both approaches were applied as implemented in MSstats and used to generate summarized intensity values for downstream statistical testing. A full description of the MSstats package is available in the paper by Kohler *et al*. (23).

## Results

### Benchmarking Dataset Characterization

**Fig. 1:**
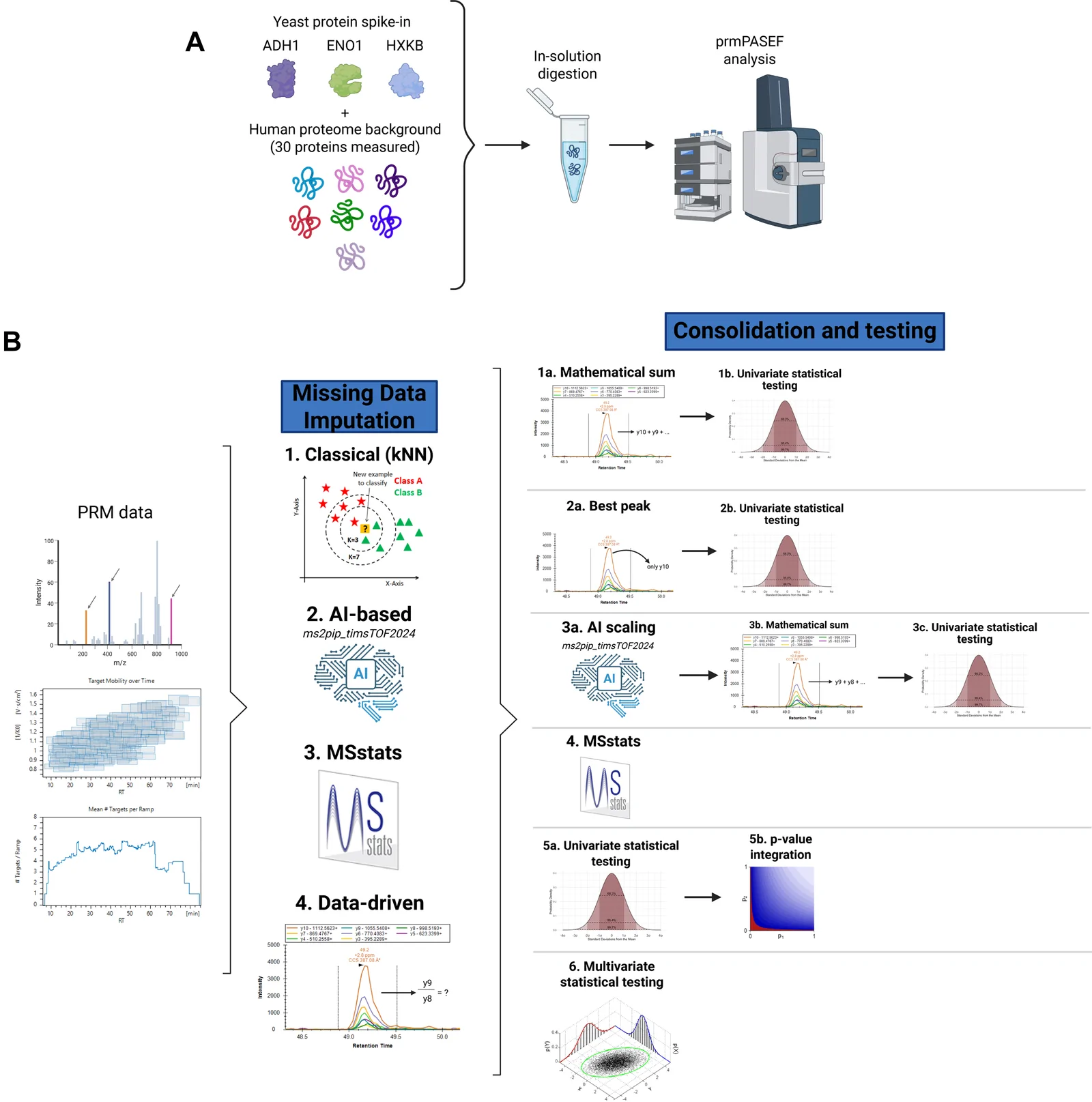
**Schematic overview of experimental design and analysis workflow.***A*, the experimental design consisted of preparing a benchmarking dataset by prmPASEF measurement of 3 spiked-in yeast proteins such as ADH1, ENO1, and HXKB at differing concentrations and 30 human proteins at a constant background level. *B*, data analysis workflows encompassed missing data imputation based of classical kNN, AI-based imputation, MSstats R library for imputation or data-driven approach. These were then followed by consolidation and testing methods such as: univariate testing for simple mathematical summation of all fragment peak areas, choosing the best representative peak, AI scaling followed by summation and univariate testing, MSstats R library, *p*-value integration or multivariate testing. AI, artificial intelligence; KNN, k-nearest neighbor.

**Fig. 2:**
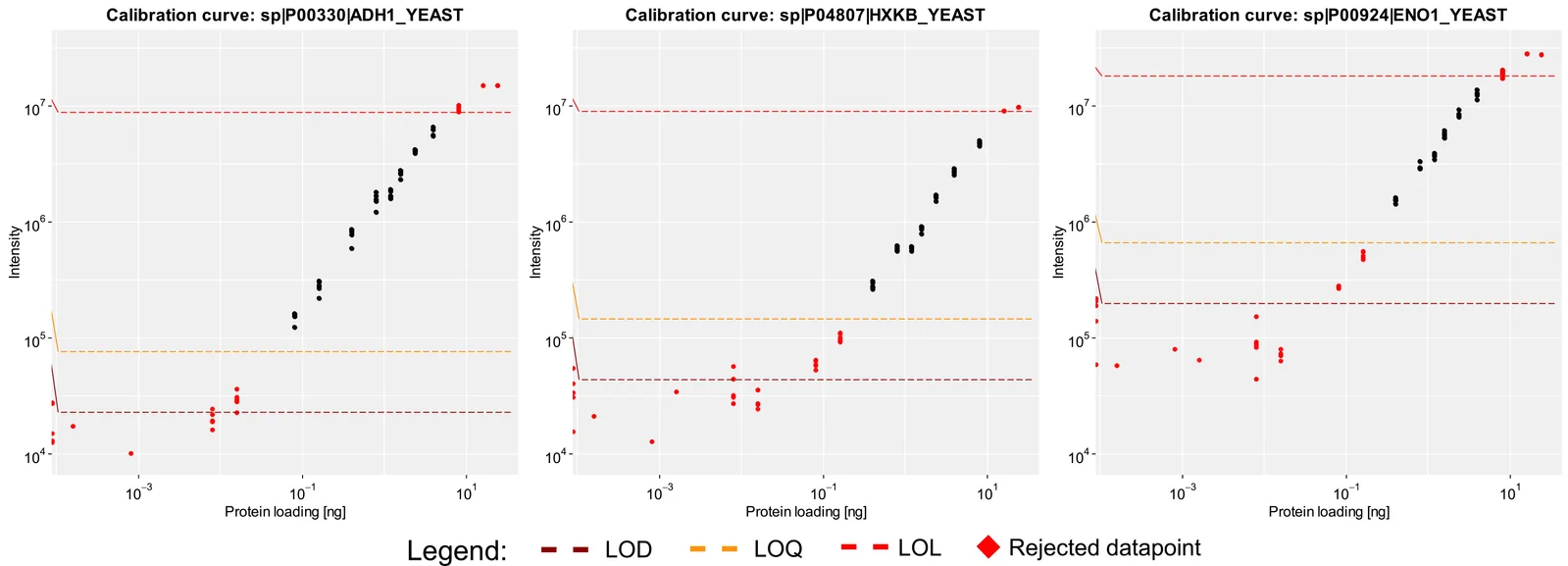
**Characterization of the benchmarking dataset.** The calibration curve for each of the spiked-in yeast proteins, that is ADH1, HXKB, and ENO1, was plotted to visualize the linear range. Limits of detection and quantification are marked as *brown* and *orange lines* respectively and correspond to the intensity calculated as a 3-fold and 10-fold value of blank samples’ standard deviation. The limit of linearity, marked in *red*, was determined using a piecewise linear approximation. Data points that were rejected from further analysis are marked in *red*.

A benchmarking dataset was prepared consisting of a constant human protein background and three yeast proteins spiked-in at a number of known concentrations in five technical replicates each (Fig. 1*A*), with further study focused on the methods of missing data imputation and statistical testing (Fig. 1*B*). First, the range of concentrations that would be characterized by a linear response was determined (Fig. 2). LOD and LOQ values for each of the spiked-in proteins were subsequently calculated based on the 3- and 10-fold standard deviations of the summed fragment peak areas for blank samples. These values were obtained after discarding any fragments that contained missing data. This resulted in identifying the lowest concentration datapoint at which the linear response was discernible, corresponding to 0.4 ng of protein per 5 μl injection. To determine the limit of linearity, the piecewise linear approximation method was employed, and an upper limit of 3.97 ng protein per 5 μl injection was established. Additional datapoints that have been characterized as being outside the linear range were rejected from further study. The final fit for the three spiked-in proteins yielded a mean coefficient of determination (r^2^) of 0.98.

### Missing Data Imputation Methods Comparison

**Fig. 3:**
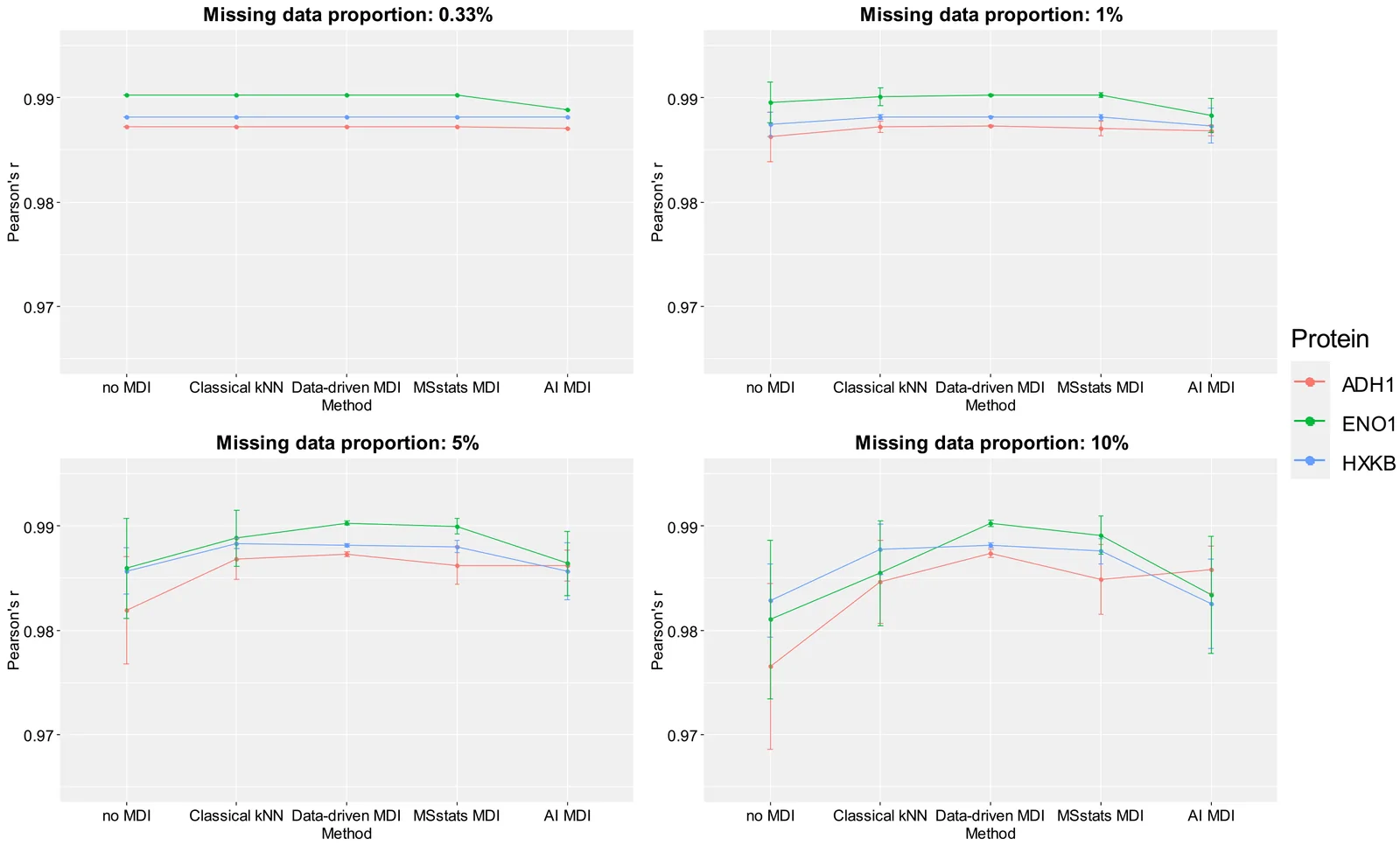
**Comparison of missing data imputation methods.** Pearson’s correlation coefficient was determined for spiked-in yeast proteins (marked *red* for ADH1, *green* for ENO1 and *blue* for HXKB) before (marked “no MDI”) and after missing data imputation by classical kNN, data-driven, MSstats and AI approaches. Since a minimal number of missing data is found in the linear response region for the spiked-in proteins, random resampling of missing data was performed for 1%, 5%, and 10% of data points. AI, artificial intelligence; KNN, k-nearest neighbor; MDI, missing-data imputation.

**Fig. 4:**
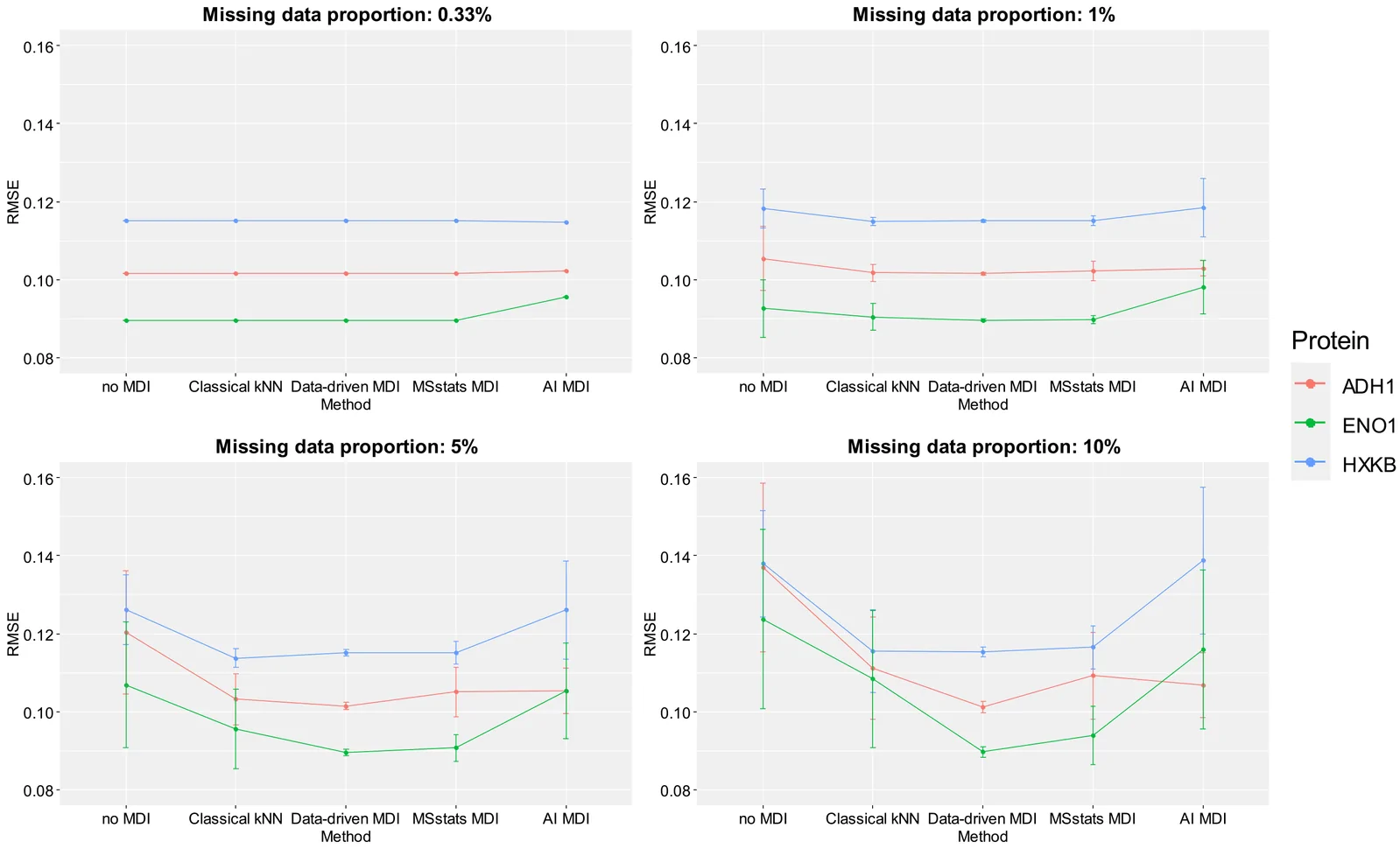
**Comparison of missing data imputation methods.** Root Mean Square Error was determined for spiked-in yeast proteins (marked *red* for ADH1, *green* for ENO1, and *blue* for HXKB) before (marked “no MDI”) and after missing data imputation by classical kNN, data-driven, and AI approaches. Since a minimal number of missing data points is observed in the linear response region for the spiked-in proteins, random resampling of missing data was performed for 1%, 5%, and 10% of the data points. AI, artificial intelligence; KNN, k-nearest neighbor; MDI, missing-data imputation.

**Supplemental Fig. S1:**
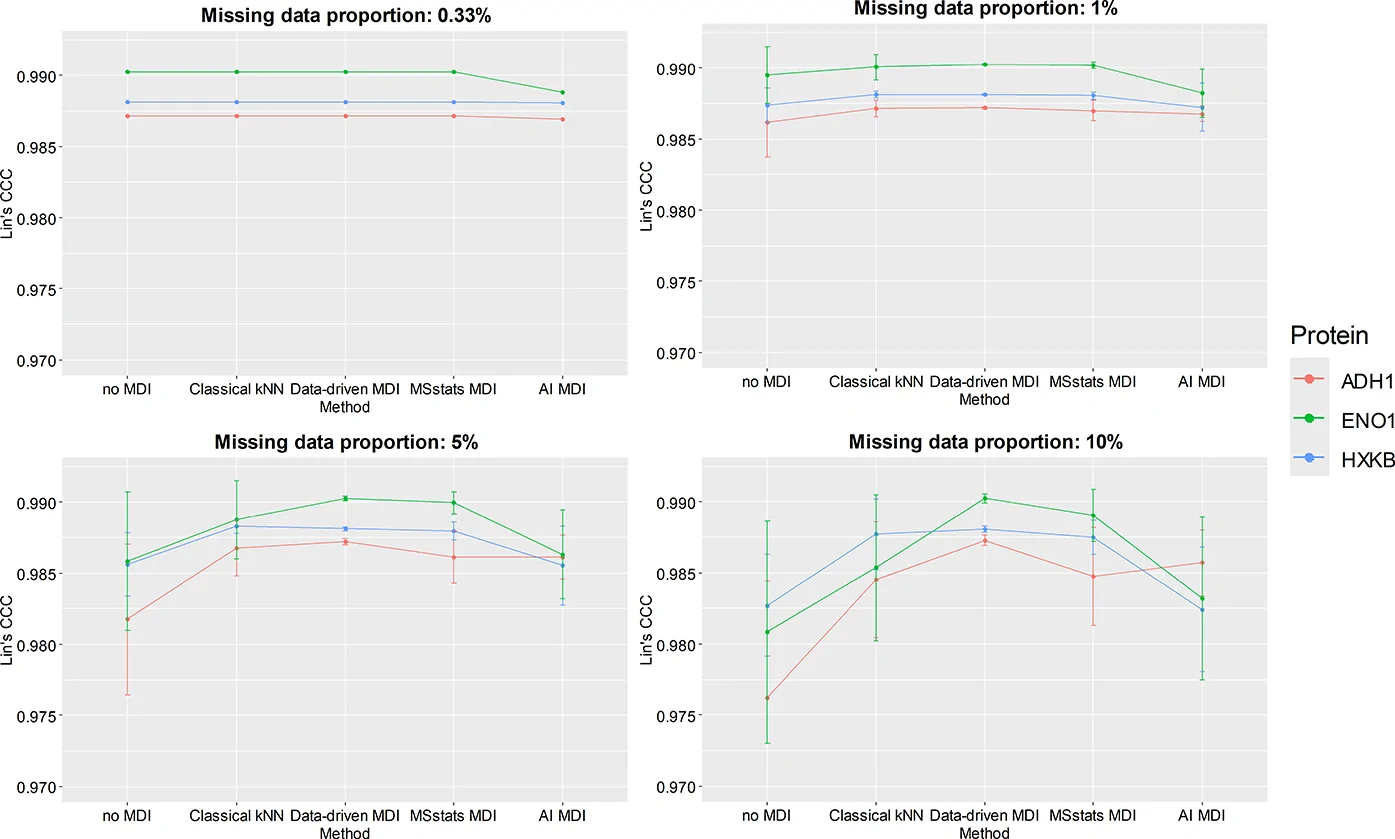


**Supplemental Fig. S2:**
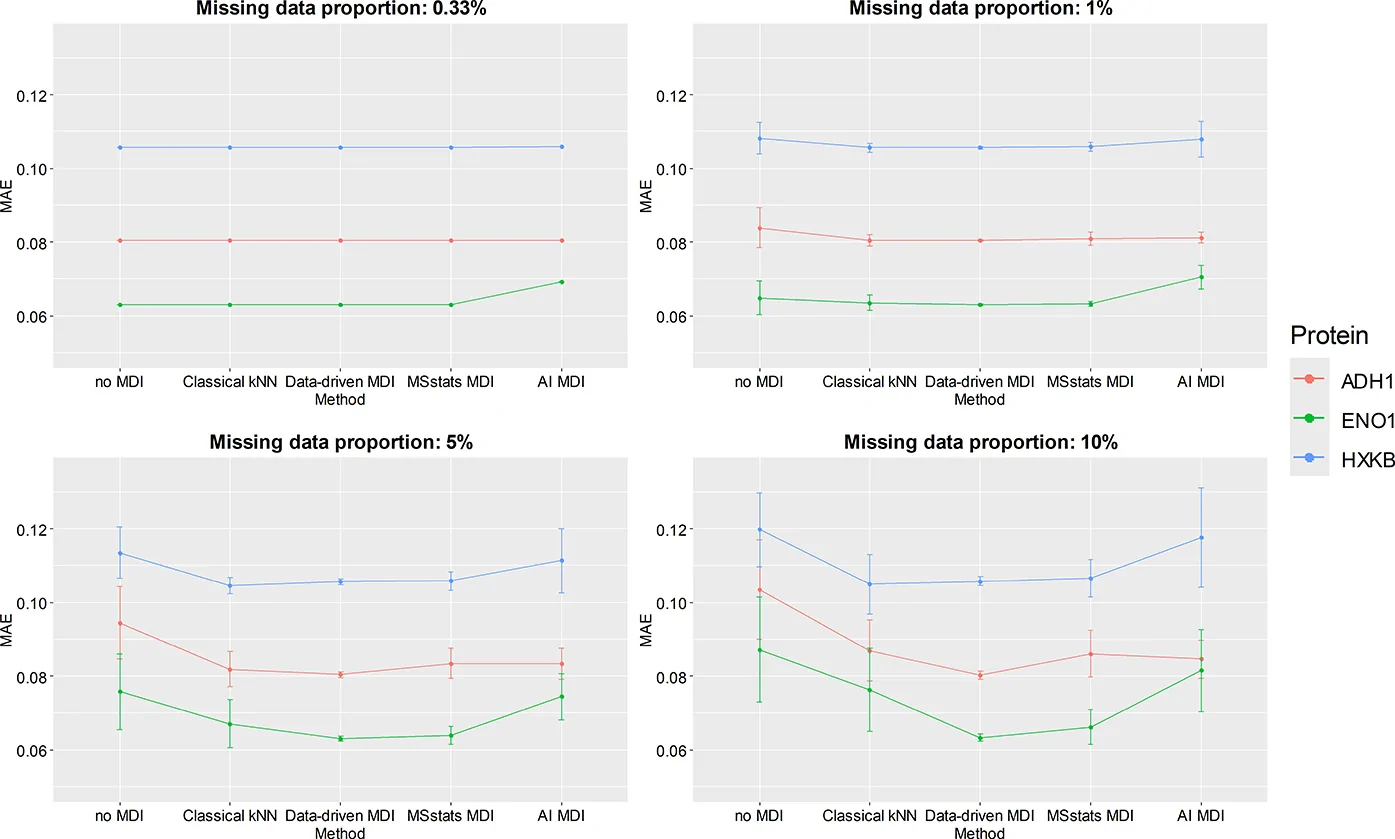


**Supplemental Fig. S3:**
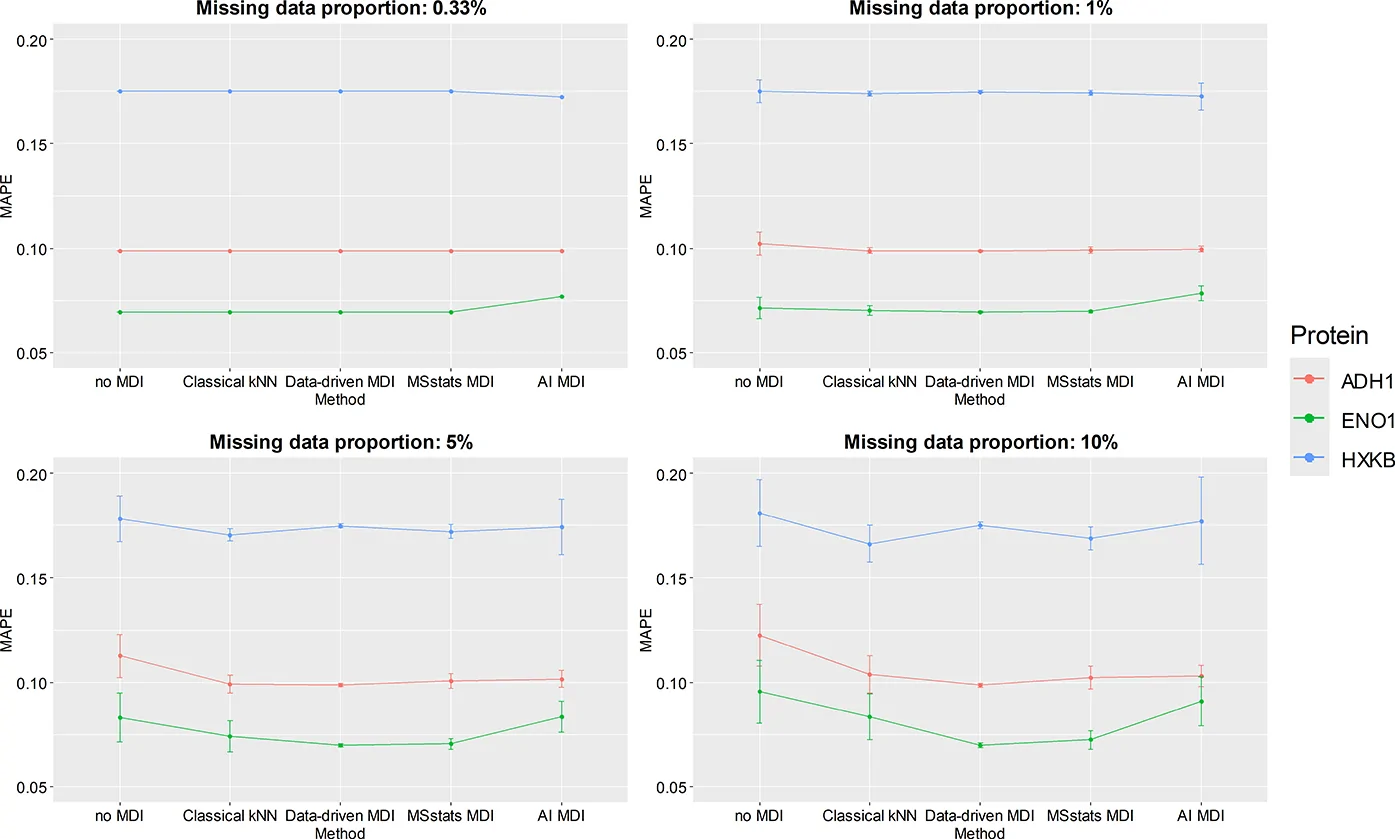


**Supplemental Fig. S4:**
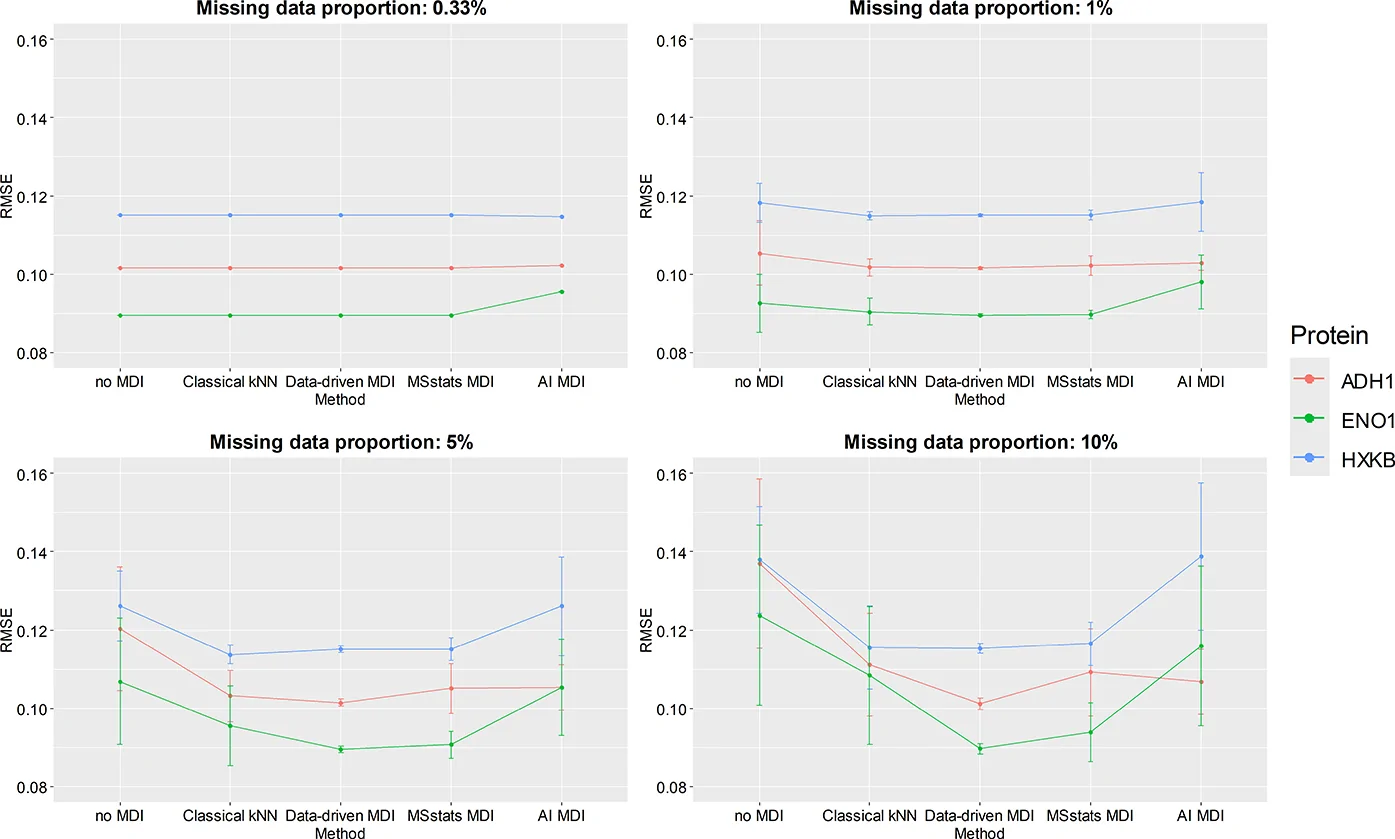


To benchmark missing data imputation approaches, datapoints in the linear response range were compared as to their Pearson’s r value or root mean square error (RMSE) before and after imputation (Figs. 3 and 4). It should be noted that a minimal amount of missing data characterizes targeted analyses, since they are performed after substantial method preparation and results are considered valid only when each peak has an acceptable signal-to-noise ratio, e.g., SNR >10 (50). Yet at the same time, no missing data can be present for further statistical testing. To visualize the performance of selected methods, such as the proposed classical kNN, data-driven, MSstats and AI scaling approaches, the original dataset with 0.33% of missing data was used. Additional datasets with 1%, 5%, or 10% randomly distributed missing values were created and imputed through a hundredfold iteration. Of all the methods, no MDI was most often characterized by the lowest Pearson’s correlation coefficient, highest RMSE and the highest variance in the resulting fit. On the other hand, data-driven missing data imputation gave, on average, the highest Pearson’s r value with minimal variance. As an example, for HXKB protein no MDI gave Pearason’s r of 0.9859 ± 0.0046, for classical kNN - 0.9888 ± 0.0025, for data-driven approach - 0.9903 ± 0.0002, for MSstats - 0.9900 ± 0.0007 and for AI-based MDI - 0.9864 ± 0.0031. Generally, the classical kNN and MSstats approaches yielded Pearson’s r and RMSE values similar to those of the data-driven approach, yet they also showed larger standard deviations, with MSstats typically exhibiting slightly lower dispersion in these metrics than classical kNN. AI MDI application had worse performance than other methods, but in the datasets with a higher amount of missing data than 0.33% (*i*.*e*., 1–10%), it gave better results than no MDI at all. Although not shown here for brevity, the same basic conclusions could be drawn when other metrics for comparison were used, such as Lin’s Concordance Correlation Coefficient (see Supplemental Fig. S1), Mean Absolute Error (see Supplemental Fig. S2), Mean Absolute Percentage Error (see Supplemental Fig. S3), Root Mean Square Error (see Supplemental Fig. S4) or Coefficient of Determination (see Supplemental Fig. S5).

### Comparison of Data Consolidation and Statistical Testing Methods

**Fig 5:**
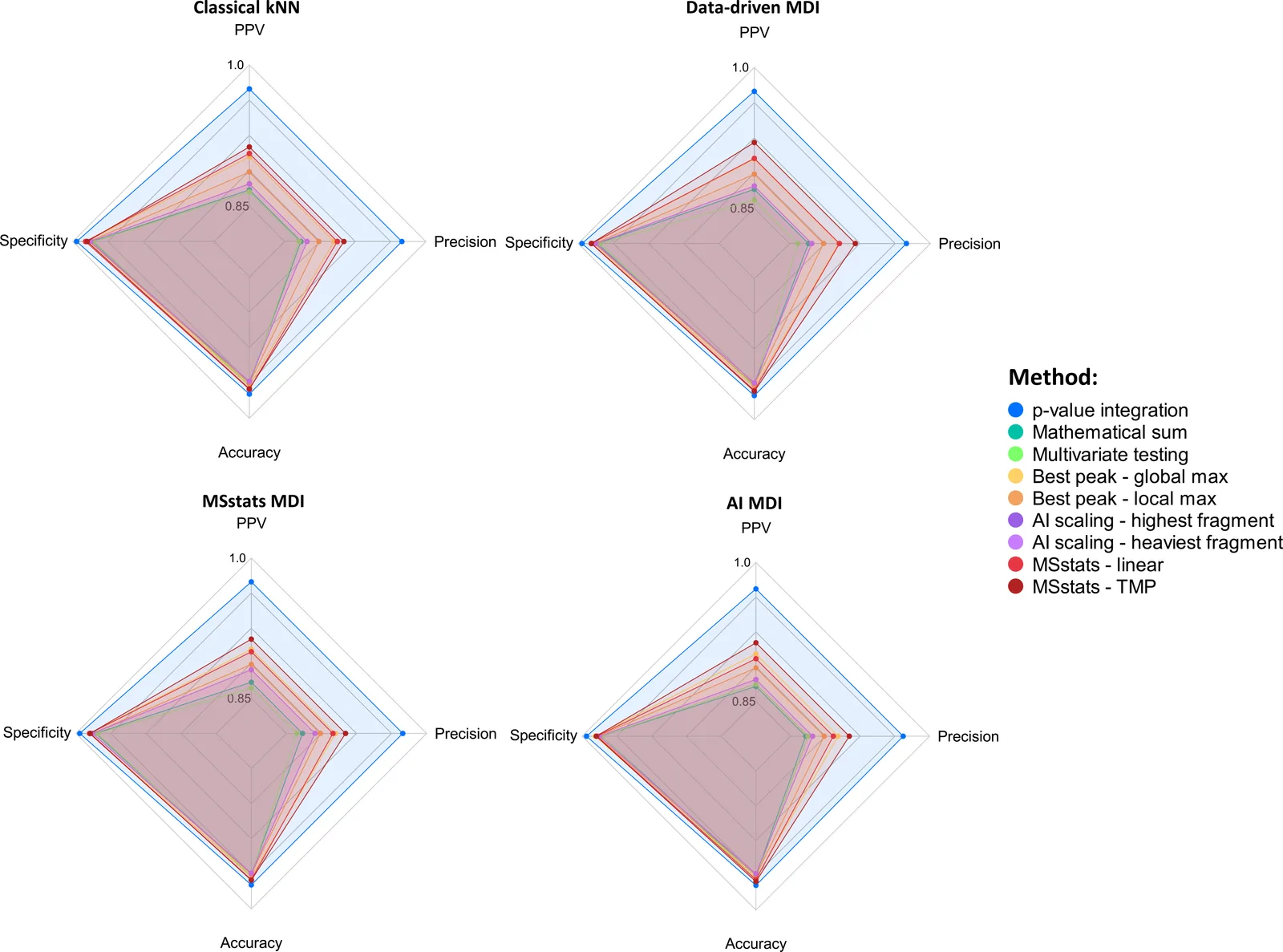
**Comparison of data consolidation and statistical testing approaches.** Parameters calculated from a confusion matrix, that is PPV, precision, accuracy, and specificity, are plotted for a number of datasets created with differing MDI methods, that is classical kNN, data-driven, MSstats, and AI approaches. Colors used are: *blue*–*p*-value integration; teal–mathematical summation; *green*–multivariate testing; *yellow*–best peak selection by global maximum; *orange*–best peak selection by local maximum; *purple*–AI scaling with highest intensity fragment as a reference; *pink*–AI scaling with heaviest intensity fragment as a reference; *red*–MSstats with linear model; *brown*–MSstats with Tukey’s Median Polish model. AI, artificial intelligence; KNN, k-nearest neighbor; MDI, missing-data imputation.

Since the datasets consist of samples with spiked-in yeast proteins and 30 background human proteins at a constant concentration, it was possible to pair groups of samples and benchmark data consolidation and statistical testing methods based on parameters derived from a contingency table (Fig. 5). This was performed on datasets after MDI (classical kNN, data-driven, MSstats and AI-based approaches), since for testing purposes no missing data can be present. Proposed approaches of data consolidation and statistical testing included: mathematical summation, best peak selection, AI scaling and univariate testing, MSstats linear or TMP models, *p*-value integration and multivariate testing (Fig. 1*B*). It should be noted that the performance of these methods is roughly similar in all cases, with the positive predictive value hovering around 90% (*i*.*e*., its compliment - FDR being around 10%). Out of the proposed approaches, Brown’s *p*-value integration can be characterized by the highest PPV with the highest accuracy, specificity, and precision. Mathematical summation, depending on the group pairing, often produced the worst results among all the tested methods in terms of the compared parameters. MSstats TMP model gave second best results, after *p*-value integration, when it comes to the compared metrics with its linear model often in third place (depending on the MDI method used). Although AI-based scaling leverages models trained on large external datasets and may, in principle, reduce fragment peak area measurement error, in this dataset and workflow it did not provide significantly improved performance compared to the other methods evaluated here, including mathematical summation, best peak selection, MSstats approaches, *p*-value integration and multivariate testing. The best peak selection, whether by global or local maximum, was also characterized by performance similar to that of AI scaling. All of the above was observed regardless of the missing data imputation approach used. Multivariate testing and *p*-value integration were the two methods that output a single *p*-value, yet no concise abundance for peptides/proteins is produced. From a practical standpoint, if these approaches were to be used, other methods would have to be utilized for data visualization or fold-change comparison. Moreover, not all of these methods are equal when it comes to their computational complexity – mathematical summation can be easily and quickly used due to its simplicity, whereas AI-based methods, especially their training, is a task that requires significant amount of computational power. A good compromise in terms of complexity, yet with extreme ease of use as a ready-made solution, was MSstats, which provided good results in most metrics.

## Discussion

Mathematical summation remains the most widely used strategy for deriving peptide- and protein-level abundance estimates in targeted PRM analyses (45). Although this approach performs adequately when SIL peptide standards are available, and absolute quantification is the primary objective, its limitations become apparent in label-free experiments, where maximizing statistical power and minimizing false discoveries are essential. The rapid expansion of global proteomics has led to an increase in the number of candidate biomarkers emerging from discovery workflows, thereby intensifying the need for scalable validation strategies (51). Classical validation methods, such as Western blotting or functional assays, are impractical when dozens or hundreds of proteins require verification (52, 53, 54). In this context, label-free targeted PRM guided by global datasets provides a practical and cost-efficient solution (14), and multiple studies have demonstrated its utility as a validation tool (55, 56, 57, 58, 59). However, the absence of systematically benchmarked data-analysis workflows remains a key limitation.

Using a dedicated benchmarking dataset, we systematically evaluated missing data imputation, data consolidation, and statistical testing methods tailored to label-free PRM analysis. In the case of MDI, a data-driven approach exploiting a constant ratio between peptide fragment signals yielded the highest accuracy and lowest variance, outperforming kNN, MSstats and AI-based methods. It should be noted that targeted analyses are unique when compared to global approaches, in the sense that missing values are uncommon, as an optimized targeted method would not select for peptides that are undetectable. Targeted workflows also benefit from recent advances in automated transition refinement strategies. For example, tools such as Avant-garde enable data-driven optimization of fragment ion selection prior to complete data acquisition and can automatically replace problematic transitions affected by interference or poor quantitative behavior (21). These operations reduce data missingness and mitigate the impact of such transitions on downstream analysis, representing an important development in refining targeted proteomics workflows.

An important limitation of the present framework is that it does not explicitly address peptide-level missingness, that is, situations in which the complete transition group of a peptide is absent from a given sample. Such events may be the effects of different sources, distinct from transition-level incompleteness, including intensity below the detection threshold, retention-time offset in scheduled acquisition, or lack of the peptide signal to record. These possibilities are mechanistically different from the loss or exclusion of individual fragment-ion transitions and may require different handling strategies. In the current study, both the data-driven and AI-based approaches were intended primarily for cases where at least partial peptide-level information was still present, allowing inference from the remaining transitions. Therefore, the conclusions of this work should be interpreted as applying mainly to transition-level missingness within detected peptide groups, while the treatment of fully missing peptide transition groups remains an important topic for future investigation.

All of the above reflects the nature of targeted PRM data: non-observed values at the fragment level are often missing not at random - frequently falling just below LOD or LOQ. Existing imputation frameworks, largely developed for global DDA or DIA workflows, operate on peptide- or protein level intensities and thus do not fully utilize MS^2^ data, making them less suitable for targeted datasets (60, 61, 62, 63). By incorporating all available fragment ion information, the data-driven method aligns more closely with the quantitative characteristics of prmPASEF.

Importantly, zeroes and missing entries should not be used interchangeably, even though they may not be conceptually distinct categories from a general statistical perspective. Zero or close to zero values in targeted proteomics could indicate a biologically missing signal, background signal integration, or left-censored data below LOD/LOQ. Therefore, it is important to distinguish between the statistical mechanism underlying a value's occurrence and its numerical encoding in the data matrix. While some circumstances may be closer to MAR and may need a different approach, many low or zero transition values in PRM are probably compatible with MNAR behavior. All of the above is separate from the classification of zero vs #N/A that is reported by software such as Skyline (https://skyline.ms), where zero can be retained as an informative value.

For data consolidation and statistical testing, Brown’s *p*-value integration consistently demonstrated the best performance in terms of PPV, precision, specificity, and accuracy. This method, unlike classical Fisher’s integration, corrects for data dependence, which is crucial in the case of targeted proteomics analyses (64, 65). Similar benefits of Brown-based integration have been reported for global DDA workflows, as was presented by *Killinger et al* (66). Nonetheless, a limitation of *p*-value integration is the lack of a consolidated abundance value, requiring a secondary step (*e*.*g*., summation) for visualization or fold-change interpretation. MSstats, as a complete solution for proteomics data analysis, provided a good compromise in terms of results, especially with the TMP approach, although it was not optimal for targeted proteomics compared to *p*-value integration. This may reflect the fact that MSstats was designed as a broadly applicable framework for proteomics data analysis rather than specifically for fragment-level consolidation in PRM data. Yet this ready-made tool can be easily used, even without deep programming knowledge; it does not require exceptional computational power and can easily integrate with many different data formats. Conversely, simple methods such as mathematical summation or best-peak selection do not utilize the multi-fragment nature of targeted data, resulting in reduced performance. A crucial factor is that fragment-ion intensities coming from the same peptide are naturally correlated and should not be interpreted separately. Therefore, instead of being evidence based on separate fragment measurements, the multivariate analysis reported here should be viewed as a complementary picture of coordinated fragment-level activity. Taken together, these considerations support Brown’s *p*-value integration as the most appropriate statistical endpoint in our framework, particularly because it explicitly accounts for dependence among fragment-level signals. A comparative overview of strengths and limitations for each method is presented in Table 1.

**Table 1:** Comparison of the Advantages and Disadvantages of Data Consolidation and Statistical Testing Approaches

Approach	Advantages	Disadvantages
Mathematical summation	• Computationally simple	• Further statistical testing takes minimal advantage of multiple fragment ion measurements
Best peak selection	• Computationally simple	• Further statistical testing takes no advantage of multiple fragment ion measurements • Relies on other data characteristics to choose the best fragment (e.g. CV)
AI scaling	• Reduces the measurement error of fragment ions across samples • Relies on external data from multiple samples, instruments, etc.	• Does not change data structure – separate data consolidation and statistical testing have to follow (e.g., mathematical summation) • Scales data only allowed by the model (e.g., no doubly charged fragments allowed) • Assumes constant fragment ion ratio across samples • Requires choosing a reference fragment ion • Computationally complex
Multivariate testing	• Takes advantage of multiple fragment ion measurements (*i*.*e*., has increased statistical power)	• Does not provide data consolidation (e.g., no straightforward ability to compare fold changes) • Computationally complex
MSstats	• Works as a universal tool for proteomics data analysis • Is a ready-made solution requiring minimal programming knowledge • Allows both missing data imputation and statistical testing in one solution • Performs well as compared to other tested methods • Can be automatically supplied with many data formats	• As a universal tool it may not be able to produce optimal results for all types of analyses and scenarios • As a ready-made solution, precise steps taken in the data analysis are partially obfuscated
*p*-value integration	• Provides best FDR, accuracy, specificity and precision of the tested methods • Takes advantage of multiple fragment ion measurements (*i*.*e*., has increased statistical power)	• Does not provide data consolidation (e.g., no straightforward ability to compare fold changes) • Has to take into account data-dependence of peptide fragment values

AI-based imputation and scaling did not provide the expected performance gains in our benchmark. Several factors may be responsible, and contribute to its performance. First of all, prmPASEF inherently produces reproducible MS2 data with a high number of points across chromatographic peak, leaving limited room for model-based improvements. Moreover, “*ms2pip_timsTOF2024”* model was optimized for global applications for tryptic peptides, but also additionally trained on immunopeptides and elastase digests, which expanded its intended purpose; however, this conflicts with our specific use case (47). Third, targeted prmPASEF may be sensitive to instrument- and method-specific details that are not captured during model training (67, 68, 69). At present, “*ms2pip_timsTOF2024”* model is most valuable during assay design e.g., transition prediction - rather than as post-acquisition correction methods for optimized targeted datasets. Although AI tools for proteomics data imputation and statistical modeling continue to evolve, current developments remain primarily focused on global acquisition strategies (43, 70). As an exception, *Park et al* developed an AI-assisted framework enabling repeatable and accurate quantification in workflows using SIL peptide standards (71). However, such solutions remain incompatible as they rely fundamentally on the presence of SIL standards and cannot be applied in the label-free validation approach.

One has to acknowledge that our results have been obtained using only a specific matrix (*i*.*e*., cancer tissue) with a singular instrument and technology (*i*.*e*., prmPASEF using timsTOF Pro) based on only 3 spiked-in and 30 background proteins. Relatively small number of proteins included in this study, along with a limited set of true positives, in our opinion reflects the practical limitations of targeted PRM experiments and available instrument throughput. While this restricts statistical power and the ability to resolve subtle performance differences, it is representative of most targeted proteomics studies focused on high-confidence quantification. The comparison is nonetheless instructive in this controlled environment since all approaches were examined using the same data, making it possible to identify method-specific trends even when total performance was comparable. All of the above was measured in a single batch without biological variability and with low number of missing values due to selection of well quantifiable peptides, all to minimize the number of confounding variables. Moreover, at present none of the available AI models have been trained to achieve extreme precision necessary for targeted proteomics, but to aid in method optimization and global analyses.

In summary, our findings demonstrate that data-driven missing data imputation followed by Brown’s *p*-value integration offers several advantages for analyzing label-free prmPASEF datasets over other benchmarked methods. This pipeline outperformed commonly used approaches, including mathematical summation, and provides a principled framework for quantitative validation in targeted proteomics. The future development of AI models explicitly tailored to the constraints and strengths of targeted proteomics represents a promising direction.

## Data Availability

Mass spectrometry data are publicly available on ProteomeXchange via the PRIDE partner repository under the identifier PXD071000 (72, 73). Skyline result file is publicly available at Panorama Public database (panoramaweb.org/prmPASEF_data_analysis_workflow_selection.url) (74). The R source code used in this study is available on GitHub (https://doi.org/10.5281/zenodo.20135373).

## Supplemental Data

This article contains supplemental data (46), including: Supplemental Table S1–The transitions monitored for each analyte; Supplemental Table S2 - Quantitative data for all transitions as input for data analysis steps performed in the study; Supplemental Note 1 - Expanded description of the data-driven missing data imputation algorithm; Supplemental Figure S1–Additional comparison of missing data imputation methods based on Lin’s Concordance Correlation Coefficient; Supplemental Figure S2–Additional comparison of missing data imputation methods based on Mean Absolute Error; Supplemental Figure S3–Additional comparison of missing data imputation methods based on Mean Absolute Percentage Error; Supplemental Figure S4–Additional comparison of missing data imputation methods based on Root Mean Square Error; Supplemental Figure S5–Additional comparison of missing data imputation methods based on Coefficient of Determination.

## Conflicts of Interest

The authors declare no competing interests.

## Figures and Tables

**Supplemental Fig. S5:**