# Supplementary material for: Label-Free Targeted Proteomics Data Analysis Workflow Selection – Benchmarking AI-based and Data-Driven Approaches
Source: Mol Cell Proteomics. 2026 Jul 23;25(8):101631. doi: 10.1016/j.mcpro.2026.101631 (PMC13520092; doi:10.1016/j.mcpro.2026.101631)
Supplement: Supplementary file 1 [file mmc3.docx]

**Label-Free Targeted Proteomics Data Analysis Workflow Selection – Benchmarking AI‑based and Data-Driven Approaches**

Daniel Fochtman^1^, Łukasz Marczak^1^, Monika Pietrowska^2^, Joanna Polańska^3^, Anna Wojakowska^1*^

*^1^ Laboratory of Mass Spectrometry, Institute of Bioorganic Chemistry Polish Academy of Sciences, Poznan, Poland*

*^2^ Center for Translational Research and Molecular Biology of Cancer, Maria Sklodowska-Curie National Research Institute of Oncology, Gliwice Branch, Gliwice, Poland*

*^3^ Department of Data Science and Engineering, Silesian University of Technology, Gliwice, Poland.*

^*^Corresponding author, e-mail: astasz@ibch.poznan.pl

**SUPPLEMENTARY NOTE 1**

**Expanded description of the data-driven missing data imputation algorithm**

|  | | | Sample 1 | Sample 2 | Sample 3 | Sample 4 |
| --- | --- | --- | --- | --- | --- | --- |
| Protein A | Peptide a | y1 | 1367.11 | 4505.61 | 6824.12 | 6166.68 |
|  |  | y2 | 763.52 | 2734.77 | 4903.03 | 8228.08 |
|  |  | y3 | **NA** | 241.48 | 323.54 | 335.84 |

The data-driven missing data imputation (MDI) algorithm relies on an assumption that relations between peak areas of different fragments for a selected peptide remain constant (1). For the purposes of this description a simplified case of a single protein with single peptide and three fragments (y1 to y3) is shown:

In the first step, all possible fragment combinations are found and ratios of peak areas in these combinations are calculated across samples. The table of raw values is transformed into a large table of ratios. For the missing values, ratios cannot be derived and the table of ratios can contain non-assigned values. Thus, for three fragments this results in ratios of ${y_{1}}/{y_{2}}$, ${y_{1}}/{y_{3}}$and ${y_{2}}/{y_{3}}$. Calculated across samples the resulting table of ratios looks like:

| Ratio | Sample 1 | Sample 2 | Sample 3 | Sample 4 |
| --- | --- | --- | --- | --- |
| ${y_{1}}/{y_{2}}$ | 1.8 | 1.6 | 1.4 | 0.7 |
| ${y_{1}}/{y_{3}}$ | - | 18.7 | 21.1 | 18.4 |
| ${y_{2}}/{y_{3}}$ | - | 11.3 | 15.2 | 24.5 |

| Ratio | Sample 1 | Sample 2 | Sample 3 | Sample 4 |
| --- | --- | --- | --- | --- |
| ${y_{1}}/{y_{2}}$ | ~~1.8~~ | ~~1.6~~ | ~~1.4~~ | ~~0.7~~ |
| ${y_{1}}/{y_{3}}$ | - | 18.7 | 21.1 | 18.4 |
| ${y_{2}}/{y_{3}}$ | - | 11.3 | 15.2 | 24.5 |

Now to calculate the missing value one can utilize any of the fragment ratios that contain information about the missing fragment. In case of this example, unknown value for fragment $y_{3}$ in Sample 1 can be calculated using only ${y_{1}}/{y_{3}}$and ${y_{2}}/{y_{3}}$, with the ${y_{1}}/{y_{2}}$ ratio being uninformative:

| Ratio | CV | Sample 1 | Sample 2 | Sample 3 | Sample 4 |
| --- | --- | --- | --- | --- | --- |
| ${y_{1}}/{y_{2}}$ | ~~33.03%~~ | ~~1.8~~ | ~~1.6~~ | ~~1.4~~ | ~~0.7~~ |
| ${y_{1}}/{y_{3}}$ | 7.73% | - | 18.7 | 21.1 | 18.4 |
| ${y_{2}}/{y_{3}}$ | 39.88% | - | 11.3 | 15.2 | 24.5 |

The selection of the best ratio to be used has to be performed when more than one ratio is available for the re-calculation of the missing value. Thus, all ratios are assigned a metric that ranks them from best to worst – in this case a coefficient of variation (CV) is utilized. For this example, the resulting table would look like:

Now that the available ratios can be ranked, one can notice that the coefficient of variation for the ${y_{1}}/{y_{3}}$ ratio is much lower than for the ${y_{2}}/{y_{3}}$ ratio (7.73% versus 39.88%). Thus, for the re‑calculation of the missing value, the ${y_{1}}/{y_{3}}$ ratio will be utilized. To proceed, a median value of ${y_{1}}/{y_{3}}$ ratios across samples is derived:

| Ratio | CV | Sample 1 | Sample 2 | Sample 3 | Sample 4 |  |
| --- | --- | --- | --- | --- | --- | --- |
| ${y_{1}}/{y_{2}}$ | ~~33.03%~~ | ~~1.8~~ | ~~1.6~~ | ~~1.4~~ | ~~0.7~~ |  |
| ${y_{1}}/{y_{3}}$ | 7.73% | - | 18.7 | 21.1 | 18.4 | $\to\tilde{{y_{1}}/{y_{3}}}=18.7$ |
| ${y_{2}}/{y_{3}}$ | ~~39.88%~~ | ~~-~~ | ~~11.3~~ | ~~15.2~~ | ~~24.5~~ |  |

Now, with a simple rearrangement a re-calculation of the missing value for $y_{3}$ can be performed:

$$\left\{ \begin{aligned} \tilde{{y_{1}}/{y_{3}}}=18.7 \\ y_{1}=1367.11 \end{aligned} \right.\to y_{3}=73.27$$

|  | | | Sample 1 | Sample 2 | Sample 3 | Sample 4 |
| --- | --- | --- | --- | --- | --- | --- |
| Protein A | Peptide a | y1 | 1367.11 | 4505.61 | 6824.12 | 6166.68 |
|  |  | y2 | 763.52 | 2734.77 | 4903.03 | 8228.08 |
|  |  | y3 | **73.27** | 241.48 | 323.54 | 335.84 |

This results in filling-out the table of raw values with missing data, that is:

**REFERENCES**

1.	MacCoss, M. J., Toth, M. J., and Matthews, D. E. (2001) Evaluation and Optimization of Ion-Current Ratio Measurements by Selected-Ion-Monitoring Mass Spectrometry. *Anal. Chem.* 73, 2976–2984
